# Supplementary material for: Heat Capacities of L-Cysteine, L-Serine, L-Threonine, L-Lysine, and L-Methionine
Source: Molecules. 2023 Jan 3;28(1):451. doi: 10.3390/molecules28010451 (PMC9823850; doi:10.3390/molecules28010451)
Supplement: Supplementary file 1 [file molecules-28-00451-s001.zip › molecules-2094924-supplementary.pdf]

Supplementary Materials

Heat capacities of L-cysteine, L-serine, L-threonine, L-lysine, and L-methionine

Václav Pokorný[1,2], *Vojtěch Štejfa*[1], *Jakub Havlín*[3], *Michal Fulem*[1], *Květoslav Růžička*[1,*]

[1] Department of Physical Chemistry, University of Chemistry and Technology, Prague, Technická 5, CZ-166 28 Prague 6, Czech Republic
[2] Institute of Macromolecular Chemistry, Czech Academy of Sciences, Heyrovského nám. 2, CZ-162 06 Prague 6, Czech Republic
[3] Central Laboratories, University of Chemistry and Technology, Prague, Technická 5, CZ-166 28 Prague 6, Czech Republic
[*] Correspondence: ruzickak@vscht.cz

Supplementary materials contain:

1) Analysis of adiabatic data for amino acids reported by the Lomonosov Moscow State University.
2) Experimental heat capacity data of L-cysteine, L-serine, L-threonine, L-lysine, and L-methionine measured using SETARAM μDSC IIIa, SETARAM MicroCalvet, PerkinElmer DSC 8500, and Quantum Design PPMS.
3) Tabulated thermodynamic functions (heat capacity, entropy, enthalpy, Gibbs energy) of L-cysteine, L-serine, L-threonine, L-lysine, and L-methionine.

1) Analysis of adiabatic data for amino acids reported by the Lomonosov Moscow State University

Adiabatic small-volume calorimeter at the Lomonosov Moscow State University was commissioned in 1997 [1] and its function was checked using copper with deviations less than 0.6 percent and 0.2 percent below and above 70 K, respectively. For *n*-heptane, deviations were below 1.4 percent and 0.3 percent [1]. In the case of recent work reporting the heat capacities of amino acids, it appears that this apparatus had a problem when switching from helium to nitrogen cooling. For L-tryptophan [2] and L-threonine [3], there is an apparent discontinuity in heat capacity near 80 K where the transition from helium cooling to nitrogen cooling occurs (see Figure S1a and S1b). In the case of L-tryptophan [2], the authors suggested that this mismatch is a phase transition. Cole et al. [4] published heat capacity of L-tryptophan previously and did not observe any such phase transition (note that agreement between Cole et al. [4] and our data [5] is excellent, as seen in Figure S1a). The same discontinuity appears for L-threonine at the same temperature as well (Figure S1b). Additional breaks on the heat capacity curve are observed around 300 as well as around 350 K for these two compounds. This problem seems to appear only in publications [2,3] and is not present in the most recent publication reporting data for L-asparagine monohydrate [6]; however, when we compare data this data with the work of Hutchens et al. [7], mutual agreement is worse than expected for adiabatic calorimeters, especially below 80 K (see Figure S1c and S1d). Based on these findings, the data published by Lukyanova et al. [3] were excluded from the heat capacity fitting for L-threonine, despite the moderate agreement with the data of this work.

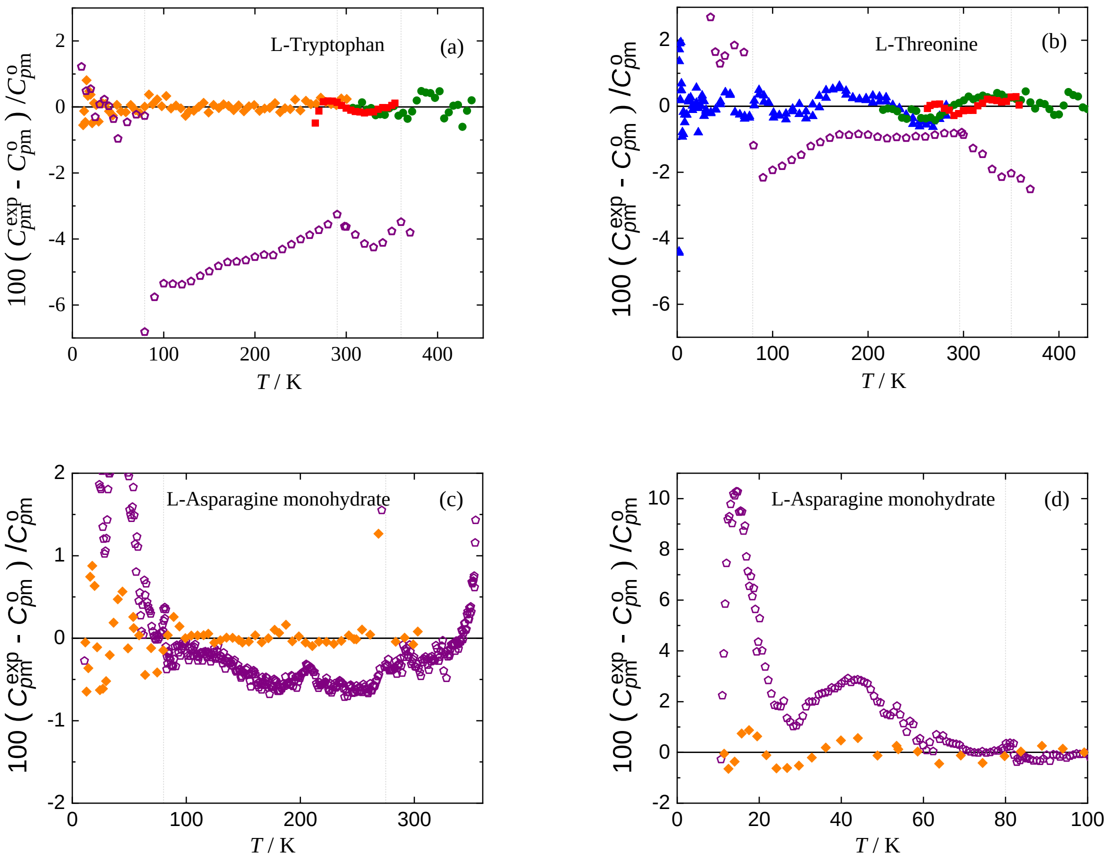

Figure S1. Relative deviations of individual experimental heat capacities from values calculated by means of Eqs. 1 and 2 based on data represented by filled symbols. (a) L-tryptophan, (b) L-threonine, (c,d) L-asparagine monohydrate. Blue ▲, this work (relaxation calorimetry); red ■, this work (Tian-Calvet calorimetry); green ●, this work (power compensation DSC); orange ◆, Hutchens and coworkers [4,7]; purple ⬠, Lukyanova and coworkers [2,3,6]. Vertical lines highlight anomalies on heat capacity data.

2) Experimental heat capacities

This section contains experimental heat capacity data obtained in this work by the means of Tian-Calvet calorimetry (2 apparatuses – SETARAM μDSC IIIa and SETARAM MicroCalvet), power-compensation differential scanning calorimetry (apparatus PerkinElmer DSC 8500), and relaxation calorimetry (apparatus Quantum Design PPMS). Due to their lower accuracy, data obtained using the PerkinElmer DSC 8500 and Quantum Design PPMS were slightly adjusted to agree with results from the more accurate SETARAM μDSC IIIa, as described in section 3.4 of the main manuscript. These correction factors are shown in the footnote of each table.

Table S1: Experimental Heat Capacity C^{o}_{pm} of L-Cysteine[a] (J K^{-1} mol^{-1}) at p = (100 ± 5) kPa.

SETARAM μDSC IIIa[b] (m = 421.93 mg)			PerkinElmer DSC 8500[c] (m = 21.23 mg)			
T / K	C^{o}_{pm} / J·K^{-1}·mol^{-1} [d]	δ_{rel} [e]	T / K	C^{o}_{pm} / J·K^{-1}·mol^{-1} [d]	C^{o}_{pm} corrected [f]	δ_{rel} [e]
267.26	132.44	-0.72	215.78	115.31	115.36	0.51
270.00	133.75	-0.48	220.72	117.06	117.11	0.47
275.00	135.88	-0.24	225.67	118.70	118.75	0.33
280.00	137.79	-0.17	230.64	120.23	120.28	0.10
285.00	139.68	-0.12	235.61	122.05	122.11	0.13
290.00	141.57	-0.05	240.59	123.58	123.63	-0.10
295.00	143.38	-0.06	245.56	125.60	125.65	0.08
300.00	145.11	-0.11	250.55	127.44	127.50	0.11
305.00	146.89	-0.13	255.54	128.98	129.03	-0.10
310.00	148.72	-0.12	260.54	130.81	130.87	-0.08
315.00	150.58	-0.08	265.53	132.46	132.51	-0.20
320.00	152.38	-0.08	270.51	134.96	135.02	0.32
325.00	154.19	-0.08	275.49	136.41	136.47	0.06
330.00	156.03	-0.05	280.47	137.90	137.96	-0.17
335.00	157.86	-0.03	285.45	139.36	139.42	-0.41
340.00	159.68	-0.02	290.43	141.32	141.38	-0.30
345.00	161.53	0.02	295.41	143.09	143.15	-0.32
350.00	163.34	0.04	300.39	145.27	145.33	-0.06
353.15	164.44	0.02	305.37	146.95	147.01	-0.14
			310.36	148.72	148.78	-0.16
			315.35	150.44	150.50	-0.21
			320.32	152.52	152.59	-0.02
			325.30	154.12	154.18	-0.15
			330.28	155.79	155.86	-0.22
			335.26	157.40	157.47	-0.34
			340.24	158.56	158.63	-0.73
			345.22	161.47	161.54	-0.02
			350.21	163.00	163.07	-0.18
			355.19	165.24	165.31	0.11
			360.18	166.79	166.86	-0.02
			365.16	168.59	168.66	0.00
			370.15	170.15	170.23	-0.12
			375.12	171.76	171.83	-0.20
			380.10	173.52	173.59	-0.18
			385.07	175.19	175.27	-0.21
			390.05	176.62	176.69	-0.38
			395.04	178.43	178.51	-0.33
			400.02	180.40	180.48	-0.18
			405.01	182.50	182.57	0.04
			409.99	185.23	185.31	0.61

SETARAM μDSC IIIa[b] (m = 421.93 mg)			PerkinElmer DSC 8500[c] (m = 21.23 mg)			
T / K	C^{o}_{pm} / J·K^{-1}·mol^{-1} [d]	δ_{rel} [e]	T / K	C^{o}_{pm} / J·K^{-1}·mol^{-1} [d]	C^{o}_{pm} corrected [f]	δ_{rel} [e]
			414.98	186.49	186.57	0.38
			419.97	188.18	188.26	0.39
			424.95	190.53	190.61	0.76
			429.93	192.49	192.57	0.92
			434.91	192.43	192.50	0.04

[a] Polymorph I_b (refcode LCYSTN12 deposited in the Cambridge structural database).
[b] Standard uncertainty of temperature is $u(T)$ = 0.05 K, and the combined expanded uncertainty of the heat capacity is $U_c(C^{o}_{pm}) = 0.01\,C^{o}_{pm}$ (0.95 level of confidence).
[c] Standard uncertainty of temperature is $u(T)$ = 0.05 K, and the combined expanded uncertainty of the heat capacity is $U_c(C^{o}_{pm}) = 0.03\,C^{o}_{pm}$ (0.95 level of confidence).
[d] Values are reported with one digit more than is justified by the experimental uncertainty to avoid round-off errors in calculations based on these results.
[e] $\delta_{rel} = 100\left(C^{o,exp}_{pm} - C^{o,calc}_{pm}\right)/C^{o,calc}_{pm}$, where $C^{o,calc}_{pm}$ is heat capacity calculated by means of Eqs. 1 and 2 with parameters from Table 5.
[f] Experimental heat capacity data from PerkinElmer DSC 8500 has been multiplied by the factor of 1.0004 to agree with the more accurate heat capacities obtained with SETARAM μDSC IIIa data.

Table S2: Experimental Heat Capacity C^{o}_{pm} of L-Serine[a] (J K^{-1} mol^{-1}) at p = (100 ± 5) kPa

SETARAM μDSC IIIa[b] (m = 423.02 mg)			PerkinElmer DSC 8500[c] (m = 22.65 mg)			
T / K	C^{o}_{pm} / J·K^{-1}·mol^{-1} [d]	δ_{rel} [e]	T / K	C^{o}_{pm} / J·K^{-1}·mol^{-1} [d]	C^{o}_{pm} corrected [f]	δ_{rel} [e]
266.15	122.67	-0.92	215.78	105.10	105.32	-1.04
270.00	124.40	-0.58	220.72	106.85	107.07	-1.00
275.00	126.38	-0.36	225.67	108.64	108.87	-0.92
280.00	128.15	-0.31	230.64	110.17	110.40	-1.08
285.00	129.83	-0.32	235.61	111.94	112.17	-1.01
290.00	131.56	-0.30	240.59	113.46	113.69	-1.17
295.00	133.27	-0.29	245.56	115.67	115.91	-0.72
300.00	134.98	-0.28	250.55	117.43	117.68	-0.67
305.00	136.70	-0.27	255.54	118.93	119.17	-0.84
310.00	138.40	-0.27	260.54	120.64	120.89	-0.82
315.00	140.13	-0.25	265.53	122.34	122.59	-0.82
320.00	141.83	-0.25	270.51	124.00	124.26	-0.83
325.00	143.58	-0.22	275.49	125.83	126.09	-0.72
330.00	145.30	-0.21	280.47	127.49	127.76	-0.73
335.00	147.14	-0.12	285.45	129.21	129.48	-0.71
340.00	148.90	-0.08	290.43	131.07	131.35	-0.57
345.00	150.67	-0.04	295.41	132.91	133.18	-0.46
350.00	152.57	0.07	300.39	134.81	135.09	-0.30
353.35	153.83	0.14	305.37	136.62	136.90	-0.21
			310.36	138.35	138.64	-0.19
			315.35	140.13	140.42	-0.13
			320.32	142.11	142.40	0.07
			325.30	143.96	144.26	0.18
			330.28	145.53	145.83	0.09
			335.26	147.31	147.62	0.14
			340.24	149.05	149.35	0.16
			345.22	150.65	150.96	0.10
			350.21	152.17	152.49	-0.02

SETARAM μDSC IIIa[b] (m = 423.02 mg)			PerkinElmer DSC 8500[c] (m = 22.65 mg)			
T / K	C^{o}_{pm} / J·K^{-1}·mol^{-1} [d]	δ_{rel} [e]	T / K	C^{o}_{pm} / J·K^{-1}·mol^{-1} [d]	C^{o}_{pm} corrected [f]	δ_{rel} [e]
			355.19	154.11	154.43	0.13
			360.18	155.29	155.61	-0.22
			365.16	157.40	157.73	0.04
			370.15	158.63	158.96	-0.26
			375.12	160.14	160.48	-0.38
			380.10	161.82	162.15	-0.40
			385.07	163.55	163.88	-0.38
			390.05	165.24	165.58	-0.39
			395.04	166.89	167.23	-0.43
			400.02	168.65	169.00	-0.39
			405.01	170.69	171.04	-0.20
			409.99	172.89	173.25	0.08
			414.98	174.72	175.08	0.14
			419.97	176.22	176.59	0.01
			424.95	177.25	177.62	-0.38
			429.93	179.46	179.84	-0.10
			434.91	181.92	182.30	0.31
			439.88	183.24	183.62	0.09
			444.86	185.14	185.52	0.18
			449.84	187.11	187.50	0.32

[a] Polymorph I (refcode LSERIN01 deposited in the Cambridge structural database).
[b] Standard uncertainty of temperature is $u(T)$ = 0.05 K, and the combined expanded uncertainty of the heat capacity is $U_c(C^{o}_{pm})$ = 0.01 C^{o}_{pm} (0.95 level of confidence).
[c] Standard uncertainty of temperature is $u(T)$ = 0.05 K, and the combined expanded uncertainty of the heat capacity is $U_c(C^{o}_{pm})$ = 0.03 C^{o}_{pm} (0.95 level of confidence).
[d] Values are reported with one digit more than is justified by the experimental uncertainty to avoid round-off errors in calculations based on these results.
[e] $\delta_{rel} = 100\left(C^{o,exp}_{pm} - C^{o,calc}_{pm}\right)/C^{o,calc}_{pm}$, where $C^{o,calc}_{pm}$ is heat capacity calculated by means of Eqs. 1 and 2 with parameters from Table 5.
[f] Experimental heat capacity data from PerkinElmer DSC 8500 have been multiplied by the factor of 1.002 to agree with the more accurate heat capacities obtained with SETARAM μDSC IIIa.

Table S3: Experimental Heat Capacity C^{o}_{pm} of L-Threonine[a] (J K^{-1} mol^{-1}) at p = (100 ± 5) kPa

SETARAM μDSC IIIa[b] (m = 426.66 mg)			PerkinElmer DSC 8500[c] (m = 21.70 mg)			
T / K	C^{o}_{pm} / J·K^{-1}·mol^{-1} [d]	δ_{rel} [e]	T / K	C^{o}_{pm} / J·K^{-1}·mol^{-1} [d]	C^{o}_{pm} corrected [f]	δ_{rel} [e]
262.26	141.10	−0.07	215.78	120.95	121.04	−0.11
265.00	142.40	0.02	220.72	123.13	123.23	−0.07
270.00	144.60	0.06	225.67	125.25	125.35	−0.08
275.00	146.76	0.07	230.64	127.30	127.40	−0.15
280.00	148.70	−0.06	235.61	129.19	129.29	−0.35
285.00	150.75	−0.12	240.59	131.28	131.38	−0.38
290.00	152.65	−0.28	245.56	133.80	133.90	−0.09
295.00	154.86	−0.22	250.55	135.87	135.98	−0.14
300.00	157.13	−0.14	255.54	137.71	137.82	−0.36
305.00	159.28	−0.12	260.54	139.83	139.94	−0.37
310.00	161.41	−0.12	265.53	142.01	142.12	−0.34
315.00	163.76	0.01	270.51	144.01	144.12	−0.42
320.00	166.02	0.09	275.49	146.33	146.45	−0.28

SETARAM μDSC IIIa[b] (m = 426.66 mg)			PerkinElmer DSC 8500[c] (m = 21.70 mg)			
T / K	C^{o}_{pm} / J·K^{-1}·mol^{-1} [d]	δ_{rel} [e]	T / K	C^{o}_{pm} / J·K^{-1}·mol^{-1} [d]	C^{o}_{pm} corrected [f]	δ_{rel} [e]
325.00	168.35	0.21	280.47	148.64	148.76	−0.16
330.00	170.44	0.19	285.45	150.94	151.05	−0.05
335.00	172.52	0.17	290.43	153.21	153.32	0.04
340.00	174.57	0.13	295.41	155.42	155.54	0.10
345.00	176.72	0.15	300.39	157.67	157.79	0.18
350.00	179.07	0.28	305.37	159.99	160.11	0.30
355.00	181.19	0.29	310.36	161.98	162.10	0.21
358.36	182.15	0.04	315.35	164.19	164.32	0.26
			320.32	166.41	166.54	0.32
			325.30	168.45	168.58	0.27
			330.28	170.50	170.63	0.23
			335.26	172.90	173.03	0.40
			340.24	174.93	175.06	0.35
			345.22	176.88	177.02	0.26
			350.21	178.99	179.13	0.26
			355.19	181.01	181.14	0.22
			360.18	183.05	183.19	0.19
			365.16	185.62	185.76	0.45
			370.15	187.09	187.23	0.12
			375.12	188.81	188.95	−0.07
			380.10	191.20	191.35	0.11
			385.07	193.18	193.33	0.06
			390.05	194.93	195.08	−0.09
			395.04	196.63	196.78	−0.26
			400.02	198.69	198.84	−0.25
			405.01	201.26	201.41	0.02
			409.99	204.10	204.26	0.43
			415.00	205.93	206.09	0.34
			419.98	207.86	208.02	0.30
			424.96	209.15	209.31	−0.03
			429.94	211.01	211.17	−0.09
			434.92	212.85	213.01	−0.14
			439.90	210.43	210.59	−2.17
			444.87	215.26	215.42	−0.81
			449.85	217.52	217.69	−0.65

[a] Polymorph I (refcode LTHREO deposited in the Cambridge structural database).
[b] Standard uncertainty of temperature is $u(T)$ = 0.05 K, and the combined expanded uncertainty of the heat capacity is $U_c(C^{o}_{pm}) = 0.01\, C^{o}_{pm}$ (0.95 level of confidence).
[c] Standard uncertainty of temperature is $u(T)$ = 0.05 K, and the combined expanded uncertainty of the heat capacity is $U_c(C^{o}_{pm}) = 0.03\, C^{o}_{pm}$ (0.95 level of confidence).
[d] Values are reported with one digit more than is justified by the experimental uncertainty to avoid round-off errors in calculations based on these results.
[e] $\delta_{rel} = 100\left(C^{o,exp}_{pm} - C^{o,calc}_{pm}\right) / C^{o,calc}_{pm}$, where $C^{o,calc}_{pm}$ is heat capacity calculated by means of Eqs. 1 and 2 with parameters from Table 5.
[f] Experimental heat capacity data from PerkinElmer DSC 8500 have been multiplied by the factor of 1.009 to agree with the more accurate heat capacities obtained with SETARAM μDSC IIIa.

Table S4 Experimental Molar Heat Capacity C^{o}_{pm} of L-Threonine[a] Obtained Using the Relaxation Technique (Quantum Design PPMS).[b] m_{THR}= 9.850 mg, m_{Cu} = 15.024 mg.

T / K	C^{o}_{pm} [c] / J·K^{-1}·mol^{-1}	δ_{rel} [d]	T/ K	C^{o}_{pm} [c] / J·K^{-1}·mol^{-1}	δ_{rel} [d]
	Run 1			Run 2	
281.865	149.64	0.03	282.030	149.24	0.03
274.871	146.17	−0.29	275.031	146.10	0.17
267.874	142.94	−0.29	268.039	142.77	−0.30
260.881	139.97	−0.39	261.035	139.89	−0.35
253.887	136.95	−0.46	254.026	136.82	−0.30
246.893	134.11	−0.63	247.024	133.93	−0.38
239.886	131.25	−0.45	240.014	131.15	−0.25
232.892	128.50	−0.56	233.004	128.50	−0.25
225.911	125.61	−0.47	226.011	125.60	−0.18
218.923	122.87	−0.61	219.010	122.75	−0.19
211.937	119.86	−0.36	212.012	119.71	0.34
204.951	116.85	−0.54	205.011	116.62	0.38
197.971	113.70	−0.25	198.009	113.65	0.42
190.990	110.59	−0.37	191.007	110.61	0.42
184.006	107.55	−0.06	184.000	107.57	0.06
176.999	104.55	−0.09	176.997	104.67	0.14
170.006	101.61	0.04	169.987	101.67	−0.10
163.018	98.406	0.00	162.994	98.414	−0.08
156.040	94.980	0.27	155.973	95.166	−0.21
149.047	91.489	0.15	148.961	91.764	−0.18
142.028	87.973	0.29	141.962	88.253	−0.15
135.027	84.581	0.14	134.962	84.734	−0.11
128.038	81.286	0.32	127.991	81.509	0.15
121.014	77.823	0.11	120.971	77.863	−0.30
114.001	73.994	0.24	113.972	74.107	0.25
107.010	70.320	0.18	106.994	70.300	0.33
100.984	66.891	0.21	100.943	66.967	0.04
95.932	64.247	0.22	95.917	64.237	0.01
90.914	61.236	0.24	90.868	61.326	−0.06
85.829	58.264	0.27	85.815	58.175	−0.79
80.780	54.720	0.35	80.751	54.778	0.56
75.753	51.167	0.47	75.734	51.129	0.11
70.694	47.638	0.55	70.677	47.588	−0.03
65.643	43.998	0.62	65.623	43.981	0.00
60.584	40.216	0.51	60.587	40.214	0.01
55.533	36.461	0.53	55.528	36.469	−0.12
50.478	32.386	0.26	50.478	32.385	0.27
45.428	28.046	0.49	45.423	28.065	0.15
40.364	23.538	−0.03	40.362	23.541	0.23
35.309	18.796	0.31	35.311	18.803	0.15
30.255	13.951	−0.30	30.254	13.956	−0.25
28.250	12.089	0.05	28.248	12.033	−0.26
26.224	10.230	−0.37	26.221	10.235	−0.49
24.205	8.4364	−0.15	24.202	8.4308	−0.25
22.186	6.7623	−0.24	22.181	6.7087	−0.17
20.151	5.2374	0.07	20.163	5.2224	−0.84
18.139	3.8210	−0.15	18.139	3.8221	−0.92
16.101	2.6198	−0.07	16.104	2.6180	−0.79
14.076	1.6663	−0.40	14.080	1.6661	0.69

T / K	C^{o}_{pm} [c] / J·K^{-1}·mol^{-1}	δ_{rel} [d]	T/ K	C^{o}_{pm} [c] / J·K^{-1}·mol^{-1}	δ_{rel} [d]
Run 1			Run 2		
12.058	0.96011	−0.23	12.061	0.96019	0.49
10.026	0.48515	−0.27	10.030	0.48581	0.48
7.936	0.20749	−0.28	7.951	0.20936	1.94
6.593	0.10963	−0.34	6.588	0.10862	0.20
5.464	0.058454	−0.20	5.463	0.058501	0.18
4.552	0.033204	0.11	4.552	0.033137	1.91
3.818	0.019222	0.10	3.818	0.019501	3.19
3.193	0.011144	0.13	3.193	0.011142	1.37
2.682	0.0067262	0.33	2.682	0.0068113	1.72
2.265	0.0040536	0.49	2.265	0.0040679	−4.40
1.919	0.0023444	0.35	1.919	0.0023434	−4.44

[a] Polymorph I (refcode LTHREO deposited in the Cambridge structural database).

[b] Standard uncertainty of temperature is $u(T)$=0.004 K, and the combined expanded uncertainty of heat capacity $U_c(C^{o}_{pm})$ with 0.95 level of confidence (k=2) is $U_c(C^{o}_{pm})$=0.1 C^{o}_{pm} below 10 K; $U_c(C^{o}_{pm})$=0.03 C^{o}_{pm} in temperature range (10 to 40) K; $U_c(C^{o}_{pm})$=0.02 C^{o}_{pm} in temperature range (40 to 300) K. Values are reported with more digits than is justified by the experimental uncertainty to avoid round-off errors in calculations based on these results. Measurements are performed in vacuum (residual pressure p<10^{-4} Pa).

[c] Experimental heat capacity data from QuantumDesign PPMS have been multiplied by the factor of 1.015 to agree with the more accurate heat capacities obtained with SETARAM µDSC IIIa data.

[d] $\delta_{rel} = 100\left(C^{o,exp}_{pm} - C^{o,calc}_{pm}\right)/C^{o,calc}_{pm}$, where $C^{o,calc}_{pm}$ is heat capacity calculated by means of Eqs. 1 and 2 with parameters from Table 5.

Table S5: Experimental Heat Capacity C^{o}_{pm} of L-Lysine (J K^{-1} mol^{-1}) at p = (100 ± 5) kPa

SETARAM MicroCalvet[a] (m = 483.6 mg)			PerkinElmer DSC 8500[b] (m = 19.29 mg)			
T / K	C^{o}_{pm} / J·K^{-1}·mol^{-1} [c]	δ_{rel} [d]	T / K	C^{o}_{pm} / J·K^{-1}·mol^{-1} [c]	C^{o}_{pm} corrected [e]	δ_{rel} [d]
	crIII			crIII		
232.00	176.25	−0.50	215.74	159.83	161.43	−2.44
235.00	178.78	−0.34	220.69	163.61	165.25	−2.17
240.00	183.41	0.07	225.64	167.61	169.29	−1.84
245.00	187.86	0.16	230.61	172.00	173.72	−1.35
250.00	192.81	0.20	235.58	176.16	177.92	−1.06
255.00	198.33	0.10	240.56	180.64	182.45	−0.71
260.00	204.83	−0.08	245.54	185.86	187.72	−0.18
265.00	212.56	−0.34	250.52	191.79	193.71	0.38
270.00	222.35	−0.48	255.51	198.14	200.12	0.67
275.00	235.39	−0.22	260.51	205.53	207.59	0.89
280.00	253.14	0.72	265.50	214.03	216.17	0.92
285.00	274.84	1.59	270.48	224.52	226.77	1.00
290.00	291.80	−0.96	275.46	237.58	239.96	1.17
	crII		280.44	253.77	256.31	1.37
295.00	294.25	0.25	285.42	271.68	274.40	0.75
300.00	288.24	0.35	290.40	283.51 [f]	286.35	−3.52
305.00	283.07	−0.15		crII		
310.00	281.09	−0.30	295.38	284.47	287.31	−1.93
315.00	281.84	−0.14	300.36	281.07	283.88	−1.05
320.00	284.44	0.08	305.34	278.72	281.51	−0.64
325.00	288.44	0.26	310.33	279.02	281.81	−0.03
330.00	293.45	0.31	315.31	278.43	281.21	−0.39

SETARAM MicroCalvet[a] (m = 483.6 mg)			PerkinElmer DSC 8500[b] (m = 19.29 mg)			
T / K	C^{o}_{pm} / J·K^{-1}·mol^{-1} [c]	δ_{rel} [d]	T / K	C^{o}_{pm} / J·K^{-1}·mol^{-1} [c]	C^{o}_{pm} corrected [e]	δ_{rel} [d]
335.00	299.15	0.17	320.29	281.70	284.52	0.05
340.00	305.43	−0.16	325.26	285.87	288.73	0.28
345.00	312.72	−0.49	330.24	291.62	294.54	0.59
350.00	322.19	−0.43	335.23	297.48	300.45	0.50
355.00	335.38	0.46	340.20	304.34	307.38	0.37
360.00	354.76 [f]	2.86	345.19	312.32	315.44	0.27
			350.17	320.99	324.20	0.08
			355.16	331.22	334.53	0.10
			360.14	347.84 [f]	351.32	1.77
			365.13	408.49 [f]	412.57	15.57
			370.11	639.96 [f]	646.36	75.02
			crI			
			375.09	291.27	294.18	0.55
			380.06	291.48	294.39	−0.13
			385.04	293.24	296.17	−0.26
			390.02	295.75	298.71	−0.13
			395.00	297.43	300.40	−0.26
			399.98	300.10	303.10	−0.05
			404.97	302.16	305.18	−0.03
			409.95	304.37	307.41	0.04
			414.94	306.69	309.76	0.16
			419.93	308.50	311.58	0.12

[a] Standard uncertainty of temperature is $u(T)$ = 0.05 K, and the combined expanded uncertainty of the heat capacity is $U_c(C^{o}_{pm}) = 0.01\,C^{o}_{pm}$ (0.95 level of confidence).

[b] Standard uncertainty of temperature is $u(T)$ = 0.05 K, and the combined expanded uncertainty of the heat capacity is $U_c(C^{o}_{pm}) = 0.03\,C^{o}_{pm}$ (0.95 level of confidence).

[c] Values are reported with one digit more than is justified by the experimental uncertainty to avoid round-off errors in calculations based on these results.

[d] $\delta_{rel} = 100\left(C^{o,exp}_{pm} - C^{o,calc}_{pm}\right)/C^{o,calc}_{pm}$, where $C^{o,calc}_{pm}$ is heat capacity calculated by means of Eqs. 1 and 2 with parameters from Table 5.

[e] Experimental heat capacity data from PerkinElmer DSC 8500 has been multiplied by the factor of 1.01 to agree with the more accurate heat capacities obtained with SETARAM μDSC IIIa.

[f] Values not considered in the correlation procedure because of their vicinity to the first-order phase transition.

Table S6 Experimental Molar Heat Capacity C^{o}_{pm} of L-Lysine Obtained Using the Relaxation Technique (Quantum Design PPMS).[a] m_{LYS}= 8.858 mg, m_{Cu} = 14.287 mg.

T / K	C^{o}_{pm} [b] / J·K^{-1}·mol^{-1}	δ_{rel} [c]	T/ K	C^{o}_{pm} [b] / J·K^{-1}·mol^{-1}	δ_{rel} [c]
Run 1, crIII			Run 2, crIII		
281.586	253.48	−1.36	281.143	252.96	−0.94
274.581	231.43	−1.42	274.170	231.30	−1.01
267.571	216.09	−0.99	267.173	216.34	−0.51
260.552	205.28	−0.26	260.152	205.72	0.25
253.492	196.98	0.34	253.090	197.31	0.75
246.400	190.08	0.65	245.995	190.33	0.99
239.330	183.98	0.67	238.940	184.22	0.98
232.295	178.29	0.53	231.908	178.42	0.77
225.300	172.60	0.22	224.926	172.81	0.50

T / K	C^{o}_{pm} [b] / J·K^{-1}·mol^{-1}	δ_{rel} [c]	T/ K	C^{o}_{pm} [b] / J·K^{-1}·mol^{-1}	δ_{rel} [c]
	Run 1, crIII			Run 2, crIII	
218.314	167.40	0.09	217.948	167.52	0.31
211.311	162.17	−0.16	210.945	162.42	0.15
204.327	157.34	−0.26	203.971	157.42	−0.06
197.339	152.64	−0.35	196.996	152.86	−0.06
190.336	148.26	−0.30	190.006	148.48	−0.01
183.345	144.09	−0.15	183.022	144.36	0.17
176.366	140.15	0.11	176.061	140.34	0.38
169.363	136.06	0.25	169.054	136.32	0.58
162.392	131.75	0.20	162.100	131.87	0.43
155.291	127.26	0.06	155.001	127.45	0.34
148.379	123.02	0.02	148.095	123.25	0.34
141.391	118.84	0.09	141.114	119.00	0.36
134.383	114.43	0.01	134.111	114.49	0.21
127.424	109.65	−0.37	127.165	109.68	−0.19
120.396	104.99	−0.53	120.142	105.08	−0.29
113.399	100.40	−0.53	113.206	100.58	−0.23
100.386	91.743	−0.21	100.189	91.730	−0.07
95.345	88.287	0.03	95.156	88.515	0.45
90.271	84.752	0.38	90.081	84.737	0.54
85.238	80.786	0.37	85.052	80.743	0.50
80.260	76.460	0.03	80.212	76.541	0.19
75.170	71.883	−0.33	75.124	71.980	−0.14
70.202	67.420	−0.44	70.161	67.497	−0.28
65.128	62.591	−0.68	65.114	62.563	−0.71
60.049	57.974	−0.20	60.012	58.032	−0.04
55.015	53.106	0.25	54.983	53.156	0.41
49.979	47.659	0.10	49.951	47.707	0.27
44.932	41.948	−0.07	44.907	42.000	0.12
39.865	36.071	0.13	39.844	36.100	0.28
34.830	29.826	0.00	34.814	29.848	0.14
29.746	23.286	−0.35	29.732	23.306	−0.19
27.728	20.772	−0.09	27.718	20.785	0.04
25.732	18.255	−0.03	25.724	18.269	0.11
23.779	15.816	−0.06	23.772	15.827	0.07
21.774	13.411	0.13	21.768	13.417	0.23
19.798	11.092	−0.03	19.792	11.094	0.04
17.840	8.9306	−0.12	17.837	8.9381	0.00
15.835	6.8595	−0.33	15.831	6.8699	−0.12
13.750	4.9184	−0.42	13.750	4.9254	−0.28
11.757	3.3335	0.12	11.755	3.3370	0.27
9.221	1.7325	0.50	9.220	1.7317	0.48
7.274	0.87315	0.23	7.273	0.87347	0.31
6.062	0.50750	0.18	6.062	0.50774	0.23
5.067	0.29332	−0.07	5.067	0.29363	0.04
4.253	0.16898	0.13	4.253	0.16913	0.22
3.589	0.096499	−0.28	3.589	0.096559	−0.22

T / K	C^{o}_{pm} [b] / J·K^{-1}·mol^{-1}	δ_{rel} [c]	T/ K	C^{o}_{pm} [b] / J·K^{-1}·mol^{-1}	δ_{rel} [c]
Run 1, crIII			Run 2, crIII		
3.047	0.055367	−0.85	3.047	0.055369	−0.85
2.609	0.032659	−0.79	2.609	0.032654	−0.81
2.259	0.020143	0.19	2.241	0.019586	0.12
1.992	0.013219	1.12	1.991	0.013139	0.68

[a] Standard uncertainty of temperature is $u(T)$=0.004 K, and the combined expanded uncertainty of heat capacity $U_c(C^{o}_{pm})$ with 0.95 level of confidence (k=2) is $U_c(C^{o}_{pm})$=0.1 C^{o}_{pm} below 10 K; $U_c(C^{o}_{pm})$=0.03 C^{o}_{pm} in temperature range (10 to 40) K; $U_c(C^{o}_{pm})$=0.02 C^{o}_{pm} in temperature range (40 to 300) K. Values are reported with more digits than is justified by the experimental uncertainty to avoid round-off errors in calculations based on these results. Measurements are performed in vacuum (residual pressure p<10^{-4} Pa).

[b] Experimental heat capacity data from QuantumDesign PPMS have been multiplied by the factor of 0.993 to agree with the more accurate heat capacities obtained with SETARAM μDSC IIIa data.

[c] $\delta_{rel} = 100\left(C^{o,exp}_{pm} - C^{o,calc}_{pm}\right)/C^{o,calc}_{pm}$, where $C^{o,calc}_{pm}$ is heat capacity calculated by means of Eqs. 1 and 2 with parameters from Table 5.

Table S7: Experimental Heat Capacity C^{o}_{pm} of L-Methionine (J K^{-1} mol^{-1}) at p = (100 ± 5) kPa

SETARAM μDSC IIIa[a] (m = 577.70 mg)			PerkinElmer DSC 8500[b] (m = 20.79 mg)			
T / K	C^{o}_{pm} / J·K^{-1}·mol^{-1} [c]	δ_{rel} [d]	T / K	C^{o}_{pm} / J·K^{-1}·mol^{-1} [c]	C^{o}_{pm} corrected [e]	δ_{rel} [d]
crIV (refcode LMETON10)			crIV (refcode LMETON10)			
265.88	198.86	−1.47	215.77	155.68	154.64	0.01
270.00	206.04	−1.35	220.72	158.98	157.91	0.18
275.00	215.53	−1.41	225.67	162.10	161.01	0.14
280.00	226.39	−1.51	230.64	165.29	164.18	0.01
285.00	239.20	−1.54	235.61	169.16	168.03	0.11
290.00	254.65	−1.36	240.59	173.17	172.01	0.08
295.00	273.31	−0.98	245.57	177.72	176.53	0.08
300.00	295.51	−0.52	250.55	182.74	181.52	0.03
305.00	319.29	−0.84	255.55	187.81	186.55	−0.35
			260.54	193.37	192.07	−0.88
			265.53	200.50	199.16	−1.03
			270.52	208.35	206.95	−1.35
			275.50	217.82	216.36	−1.49
crIII (refcode LMETON13)			280.48	229.47	227.93	−1.34
310.00	321.28	0.07	285.45	243.16	241.53	−1.10
315.00	280.96	0.73	290.44	260.06	258.32	−0.49
320.00	253.95	−0.81	295.42	279.56	277.69	0.03
325.00	244.43	−0.05	300.40	303.85	301.81	0.99
330.00	241.63	0.56	305.38	327.01	324.82	0.25
335.00	241.72	0.52	crIII (refcode LMETON13)			
340.00	242.98	0.02	310.37	316.55	314.43	−0.85
345.00	244.91	−0.36	315.35	273.68	271.85	−1.79
350.00	247.66	−0.27	320.33	251.57	249.88	−2.01
353.31	249.75	−0.06	325.31	244.30	242.66	−0.60
			330.28	242.34	240.72	0.21
			335.27	242.98	241.35	0.33
			340.25	244.90	243.26	0.08
			345.23	246.92	245.27	−0.26
			350.22	249.43	247.76	−0.27
			355.20	252.76	251.07	0.12

SETARAM µDSC IIIa[a] (m = 577.70 mg)			PerkinElmer DSC 8500[b] (m = 20.79 mg)			
T / K	C^{o}_{pm} / J·K^{-1}·mol^{-1} [c]	δ_{rel} [d]	T / K	C^{o}_{pm} / J·K^{-1}·mol^{-1} [c]	C^{o}_{pm} corrected [e]	δ_{rel} [d]
			360.19	255.76	254.05	0.37
			365.17	258.82	257.09	0.58
			370.16	261.96	260.20	0.68
			375.13	264.91	263.14	0.52
			380.11	267.98	266.18	0.15
			385.08	271.74	269.92	−0.26
			390.06	277.66	275.80	−0.27
			crII (refcode LMETON14)			
			395.05	309.03 [f]	306.96	12.85
			400.03	272.99	271.16	−0.02
			405.02	272.59	270.76	0.05
			410.00	276.73 [f]	274.88	1.72
			414.99	280.55 [f]	278.67	3.19
			419.98	283.55 [f]	281.65	4.29
			crI			
			424.96	272.40	270.57	0.11
			429.94	271.95	270.13	−0.19
			434.92	272.98	271.15	−0.01
			439.89	273.52	271.69	−0.08
			444.87	273.90	272.06	−0.26
			449.85	276.24	274.39	0.21
			454.83	277.80	275.94	0.34
			459.81	278.26	276.40	0.01
			464.80	279.78	277.91	0.01
			469.78	280.95	279.07	−0.17

[a] Standard uncertainty of temperature is $u(T)$ = 0.05 K, and the combined expanded uncertainty of the heat capacity is $U_c(C^{o}_{pm}) = 0.01\,C^{o}_{pm}$ (0.95 level of confidence).

[b] Standard uncertainty of temperature is $u(T)$ = 0.05 K, and the combined expanded uncertainty of the heat capacity is $U_c(C^{o}_{pm}) = 0.03\,C^{o}_{pm}$ (0.95 level of confidence).

[c] Values are reported with one digit more than is justified by the experimental uncertainty to avoid round-off errors in calculations based on these results.

[d] $\delta_{rel} = 100\left(C^{o,exp}_{pm} - C^{o,calc}_{pm}\right)/C^{o,calc}_{pm}$, where $C^{o,calc}_{pm}$ is heat capacity calculated by means of Eqs. 1 and 2 with parameters from Table 5.

[e] Experimental heat capacity data from PerkinElmer DSC 8500 has been multiplied by the factor of 0.9933 to agree with the more accurate heat capacities obtained with SETARAM µDSC IIIa.

[f] Values not considered in the correlation procedure because of their vicinity to the first-order phase transition.

3) Tabulated thermodynamic functions

Standard thermodynamic functions were calculated using fundamental thermodynamic relationships (assuming residual entropy at 0 K for all crystalline amino acids to be 0 J·K^{-1}·mol^{-1}) and heat capacities $C^{o}_{pm}(T)$ represented by Eqs 1 and 2 using parameters listed in Table 5:

$$S^{o}_{m}(T) = \int_{0}^{T} \frac{C^{o}_{pm}(T)}{T} \mathrm{d}T \qquad \text{(S1)}$$

$$\Delta_{0}^{T} H^{o}_{m} = \int_{0}^{T} C^{o}_{pm}(T) \, \mathrm{d}T \qquad \text{(S2)}$$

$$\Delta_{0}^{T} G^{o}_{m} = \Delta_{0}^{T} H^{o}_{m} - T S^{o}_{m}(T) \qquad \text{(S3)}$$

Table S8. Standard Thermodynamic Functions of L-Cysteine in the Crystal State[a] at p = 0.1MPa.[b]

T / K	C^{o}_{pm} / J·K^{-1}·mol^{-1}	S^{o}_{m} / J·K^{-1}·mol^{-1}	$\Delta_{0}^{T} H^{o}_{m}$ / kJ·mol^{-1}	$\Delta_{0}^{T} G^{o}_{m}$ / kJ·mol^{-1}
		crystal I$_a$ (refcode LCYSTN22)[a]		
1[c]	4.184E−04	1.347E−04	1.017E−07	−3.295E−08
2[c]	3.832E−03	1.196E−03	1.818E−06	−5.740E−07
3[c]	1.467E−02	4.459E−03	1.022E−05	−3.154E−06
4[c]	3.905E−02	1.161E−02	3.565E−05	−1.078E−05
5[c]	8.474E−02	2.476E−02	9.541E−05	−2.837E−05
6	1.609E−01	4.640E−02	2.153E−04	−6.312E−05
7	2.775E−01	7.937E−02	4.307E−04	−1.249E−04
8	4.446E−01	1.267E−01	7.871E−04	−2.267E−04
9	6.714E−01	1.916E−01	1.340E−03	−3.842E−04
10	9.657E−01	2.769E−01	2.152E−03	−6.166E−04
11	1.333	3.856E−01	3.295E−03	−9.458E−04
12	1.774	5.199E−01	4.842E−03	−1.396E−03
13	2.290	6.818E−01	6.868E−03	−1.995E−03
14	2.876	8.725E−01	9.445E−03	−2.769E−03
15	3.526	1.093	1.264E−02	−3.750E−03
16	4.233	1.343	1.652E−02	−4.965E−03
17	4.990	1.622	2.112E−02	−6.444E−03
18	5.788	1.929	2.651E−02	−8.218E−03
19	6.621	2.264	3.271E−02	−1.031E−02
20	7.483	2.626	3.976E−02	−1.276E−02
25	12.18	4.789	8.866E−02	−3.106E−02
30	17.25	7.455	1.622E−01	−6.148E−02
35	22.31	10.50	2.611E−01	−1.062E−01
40	27.11	13.79	3.848E−01	−1.669E−01
45	31.58	17.25	5.317E−01	−2.444E−01
50	35.73	20.79	7.001E−01	−3.395E−01
55	39.67	24.38	8.886E−01	−4.524E−01
60	43.54	28.00	1.097	−5.833E−01
65	47.50	31.64	1.324	−7.324E−01
70	51.75	35.31	1.572	−8.998E−01

T / K	C^{o}_{pm} / J·K^{-1}·mol^{-1}	S^{o}_{m} / J·K^{-1}·mol^{-1}	$\Delta_0^T H^{o}_{m}$ / kJ·mol^{-1}	$\Delta_0^T G^{o}_{m}$ / kJ·mol^{-1}
		crystal I_b (refcode LCYSTN12)[a]		
75	57.41	39.15	1.851	-1.039
80	60.52	42.96	2.146	-1.198
85	63.23	46.71	2.455	-1.375
90	65.81	50.40	2.778	-1.571
95	68.26	54.02	3.113	-1.785
100	70.62	57.59	3.460	-2.017
110	75.07	64.53	4.189	-2.534
120	79.24	71.24	4.961	-3.120
130	83.21	77.74	5.773	-3.771
140	87.05	84.05	6.624	-4.487
150	90.81	90.18	7.514	-5.264
160	94.51	96.16	8.440	-6.102
170	98.18	102.0	9.404	-6.999
180	101.8	107.7	10.40	-7.954
190	105.5	113.3	11.44	-8.966
200	109.1	118.8	12.51	-10.033
210	112.7	124.2	13.62	-11.15
220	116.3	129.6	14.77	-12.33
230	119.9	134.8	15.95	-13.56
240	123.5	140.0	17.17	-14.84
250	127.2	145.1	18.42	-16.17
260	130.8	150.2	19.71	-17.55
270	134.4	155.2	21.03	-18.99
273.15	135.5	156.7	21.46	-19.45
280	138.0	160.1	22.40	-20.47
290	141.7	165.0	23.79	-22.00
298.15	144.6	169.0	24.96	-23.28
300	145.3	169.9	25.23	-23.58
310	148.9	174.7	26.70	-25.21
320	152.5	179.5	28.21	-26.89
330	156.2	184.2	29.75	-28.61
340	159.8	189.0	31.33	-30.38
350	163.3	193.6	32.94	-32.20
360	166.9	198.3	34.60	-34.07
370	170.5	202.9	36.28	-35.98
380	174.0	207.5	38.00	-37.94
390	177.5	212.1	39.76	-39.94
400	180.9	216.6	41.55	-41.99
410	184.4	221.1	43.37	-44.09
420	187.7	225.6	45.23	-46.23
430	191.1	230.0	47.12	-48.41

[a] Crystal structure deposited in the Cambridge Structural Database; see also Table 2 in the main article.

[b] The combined expanded uncertainty of heat capacity $U_c(C^{o}_{pm})$ as well as of all calculated thermodynamic values (with 0.95 level of confidence, k=2) is: $U_c(X)$=0.1 X below 10 K; $U_c(X)$=0.03 X in temperature range (10 to 40) K; $U_c(X)$=0.02 X in temperature range (40 to 260) K; $U_c(X)$=0.01 X in temperature range (260 to 350) K; $U_c(X)$=0.02 X in temperature range (350 to 450) K, where X represents the heat capacity or the thermodynamic property. Values are reported with one digit more than is justified by the experimental uncertainty to avoid round-off errors in calculations based on these results.

[c] Extrapolated values.

Table S9. Standard Thermodynamic Functions of L-Serine in the Crystal State[a] at p = 0.1 MPa.[b]

T / K	C_{pm}^{o} / J·K^{-1}·mol^{-1}	S_{m}^{o} / J·K^{-1}·mol^{-1}	$\Delta_0^T H_m^o$ / kJ·mol^{-1}	$\Delta_0^T G_m^o$ / kJ·mol^{-1}
1[c]	4.258E-04	1.378E-04	1.039E-07	-3.382E-08
2[c]	3.817E-03	1.204E-03	1.826E-06	-5.814E-07
3[c]	1.429E-02	4.412E-03	1.008E-05	-3.152E-06
4[c]	3.715E-02	1.129E-02	3.452E-05	-1.062E-05
5[c]	7.874E-02	2.363E-02	9.062E-05	-2.755E-05
6[c]	1.460E-01	4.349E-02	2.006E-04	-6.039E-05
7[c]	2.460E-01	7.304E-02	3.935E-04	-1.178E-04
8[c]	3.852E-01	1.145E-01	7.056E-04	-2.105E-04
9[c]	5.692E-01	1.700E-01	1.179E-03	-3.515E-04
10[c]	8.018E-01	2.416E-01	1.860E-03	-5.559E-04
11	1.085	3.309E-01	2.799E-03	-8.406E-04
12	1.419	4.393E-01	4.047E-03	-1.224E-03
13	1.803	5.677E-01	5.654E-03	-1.726E-03
14	2.232	7.168E-01	7.668E-03	-2.366E-03
15	2.704	8.866E-01	1.013E-02	-3.166E-03
16	3.214	1.077	1.309E-02	-4.147E-03
17	3.757	1.288	1.657E-02	-5.328E-03
18	4.330	1.519	2.061E-02	-6.730E-03
19	4.931	1.769	2.524E-02	-8.372E-03
20	5.556	2.038	3.048E-02	-1.027E-02
25	9.061	3.643	6.678E-02	-2.430E-02
30	13.03	5.641	1.219E-01	-4.736E-02
35	17.21	7.961	1.974E-01	-8.125E-02
40	21.44	10.54	2.940E-01	-1.274E-01
45	25.61	13.30	4.117E-01	-1.869E-01
50	29.68	16.21	5.500E-01	-2.607E-01
55	33.59	19.23	7.082E-01	-3.492E-01
60	37.33	22.31	8.856E-01	-4.530E-01
65	40.88	25.44	1.081	-5.724E-01
70	44.23	28.59	1.294	-7.075E-01
75	47.40	31.75	1.523	-8.584E-01
80	50.39	34.91	1.768	-1.025
85	53.22	38.05	2.027	-1.207
90	55.91	41.17	2.300	-1.406
95	58.47	44.26	2.586	-1.619
100	60.93	47.32	2.884	-1.848
110	65.60	53.35	3.517	-2.352
120	70.04	59.25	4.195	-2.915
130	74.29	65.03	4.917	-3.536
140	78.38	70.68	5.681	-4.215
150	82.34	76.22	6.484	-4.949
160	86.20	81.66	7.327	-5.739
170	89.96	87.00	8.208	-6.582
180	93.65	92.25	9.126	-7.479
190	97.28	97.41	10.08	-8.427
200	100.9	102.5	11.07	-9.426
210	104.4	107.5	12.10	-10.48
220	107.9	112.4	13.16	-11.58

T / K	C^{o}_{pm} / J·K^{-1}·mol^{-1}	S^{o}_{m} / J·K^{-1}·mol^{-1}	$\Delta_0^T H^{o}_{m}$ / kJ·mol^{-1}	$\Delta_0^T G^{o}_{m}$ / kJ·mol^{-1}
230	111.4	117.3	14.26	-12.73
240	114.8	122.1	15.39	-13.92
250	118.3	126.9	16.55	-15.17
260	121.7	131.6	17.75	-16.46
270	125.1	136.2	18.99	-17.80
273.15	126.2	137.7	19.38	-18.23
280	128.5	140.9	20.26	-19.18
290	132.0	145.4	21.56	-20.62
298.15	134.7	149.1	22.64	-21.82
300	135.4	150.0	22.89	-22.09
310	138.8	154.5	24.27	-23.61
320	142.2	158.9	25.67	-25.18
330	145.6	163.3	27.11	-26.79
340	149.0	167.7	28.58	-28.45
350	152.5	172.1	30.09	-30.15
360	155.9	176.5	31.63	-31.89
370	159.3	180.8	33.21	-33.68
380	162.8	185.1	34.82	-35.51
390	166.2	189.3	36.46	-37.38
400	169.7	193.6	38.14	-39.29
410	173.1	197.8	39.86	-41.25
420	176.6	202.0	41.60	-43.25
430	180.0	206.2	43.39	-45.29
440	183.5	210.4	45.20	-47.37
450	187.0	214.6	47.06	-49.50

[a] Crystal structure deposited in the Cambridge Structural Database with refcode LSERIN01 (see the section 2.2 and Table 2 in the main article).

[b] The combined expanded uncertainty of heat capacity $U_c(C^{o}_{pm})$ as well as of all calculated thermodynamic values (with 0.95 level of confidence, k=2) is: $U_c(X)$=0.1 X below 10 K; $U_c(X)$=0.03 X in temperature range (10 to 40) K; $U_c(X)$=0.02 X in temperature range (40 to 260) K; $U_c(X)$=0.01 X in temperature range (260 to 350) K; $U_c(X)$=0.02 X in temperature range (350 to 450) K, where X represents the heat capacity or the thermodynamic property. Values are reported with one digit more than is justified by the experimental uncertainty to avoid round-off errors in calculations based on these results.

[c] Extrapolated values.

Table S10. Standard Thermodynamic Functions of L-Threonine in the Crystal State[a] at p = 0.1 MPa.[b]

T / K	C^{o}_{pm} / J·K^{-1}·mol^{-1}	S^{o}_{m} / J·K^{-1}·mol^{-1}	$\Delta_0^T H^{o}_{m}$ / kJ·mol^{-1}	$\Delta_0^T G^{o}_{m}$ / kJ·mol^{-1}
1[c]	3.608E−04	1.222E−04	9.133E−08	−3.086E−08
2	2.770E−03	9.413E−04	1.405E−06	−4.773E−07
3	9.223E−03	3.113E−03	6.975E−06	−2.364E−06
4	2.207E−02	7.340E−03	2.197E−05	−7.387E−06
5	4.436E−02	1.445E−02	5.425E−05	−1.800E−05
6	8.014E−02	2.546E−02	1.152E−04	−3.759E−05
7	1.347E−01	4.163E−02	2.208E−04	−7.064E−05
8	2.145E−01	6.450E−02	3.929E−04	−1.231E−04
9	3.275E−01	9.592E−02	6.608E−04	−2.025E−04
10	4.817E−01	1.380E−01	1.062E−03	−3.185E−04
11	6.847E−01	1.930E−01	1.641E−03	−4.828E−04
12	9.413E−01	2.632E−01	2.449E−03	−7.096E−04
13	1.256	3.506E−01	3.542E−03	−1.015E−03
14	1.631	4.570E−01	4.981E−03	−1.417E−03
15	2.068	5.840E−01	6.825E−03	−1.936E−03
16	2.566	7.331E−01	9.137E−03	−2.593E−03
17	3.122	9.050E−01	1.198E−02	−3.410E−03
18	3.733	1.101	1.540E−02	−4.410E−03
19	4.394	1.320	1.946E−02	−5.619E−03
20	5.098	1.563	2.420E−02	−7.058E−03
25	9.118	3.119	5.944E−02	−1.853E−02
30	13.73	5.183	1.164E−01	−3.909E−02
35	18.54	7.660	1.971E−01	−7.105E−02
40	23.23	10.44	3.016E−01	−1.162E−01
45	27.66	13.44	4.289E−01	−1.758E−01
50	31.86	16.57	5.778E−01	−2.508E−01
55	35.91	19.80	7.473E−01	−3.417E−01
60	39.84	23.09	9.367E−01	−4.489E−01
65	43.63	26.43	1.145	−5.727E−01
70	47.28	29.80	1.373	−7.133E−01
75	50.80	33.18	1.618	−8.708E−01
80	54.19	36.57	1.881	−1.045
85	57.45	39.96	2.160	−1.237
90	60.59	43.33	2.455	−1.445
95	63.63	46.69	2.766	−1.670
100	66.56	50.03	3.091	−1.912
110	72.14	56.63	3.785	−2.445
120	77.42	63.14	4.533	−3.044
130	82.45	69.54	5.332	−3.707
140	87.28	75.82	6.181	−4.434
150	91.96	82.01	7.077	−5.223
160	96.54	88.09	8.020	−6.074
170	101.1	94.08	9.008	−6.985
180	105.5	100.0	10.04	−7.955
190	109.9	105.8	11.12	−8.984
200	114.3	111.6	12.24	−10.07

T / K	C^{o}_{pm} / J·K^{-1}·mol^{-1}	S^{o}_{m} / J·K^{-1}·mol^{-1}	$\Delta^{T}_{0}H^{o}_{m}$ / kJ·mol^{-1}	$\Delta^{T}_{0}G^{o}_{m}$ / kJ·mol^{-1}
210	118.7	117.2	13.40	−11.22
220	123.0	122.9	14.61	−12.42
230	127.3	128.4	15.86	−13.67
240	131.6	133.9	17.16	−14.98
250	135.9	139.4	18.50	−16.35
260	140.2	144.8	19.88	−17.77
270	144.5	150.2	21.30	−19.25
273.15	145.9	151.9	21.76	−19.72
280	148.8	155.5	22.77	−20.78
290	153.1	160.8	24.28	−22.36
298.15	156.6	165.1	25.54	−23.68
300	157.3	166.1	25.83	−23.99
310	161.6	171.3	27.42	−25.68
320	165.9	176.5	29.06	−27.42
330	170.1	181.7	30.74	−29.21
340	174.4	186.8	32.46	−31.05
350	178.6	191.9	34.23	−32.94
360	182.8	197.0	36.04	−34.89
370	187.0	202.1	37.88	−36.88
380	191.1	207.1	39.77	−38.93
390	195.2	212.1	41.71	−41.03
400	199.3	217.1	43.68	−43.17
410	203.4	222.1	45.69	−45.37
420	207.4	227.1	47.75	−47.61
430	211.4	232.0	49.84	−49.91
440	215.3	236.9	51.97	−52.25
450	219.2	241.8	54.15	−54.65

[a] Crystal structure deposited in the Cambridge Structural Database with refcode LTHREO (see the section 2.2 and Table 2 in the main article).

[b] The combined expanded uncertainty of heat capacity $U_c(C^{o}_{pm})$ as well as of all calculated thermodynamic values (with 0.95 level of confidence, k=2) is: $U_c(X)$=0.1 X below 10 K; $U_c(X)$=0.03 X in temperature range (10 to 40) K; $U_c(X)$=0.02 X in temperature range (40 to 260) K; $U_c(X)$=0.01 X in temperature range (260 to 350) K; $U_c(X)$=0.02 X in temperature range (350 to 420) K, where X represents the heat capacity or the thermodynamic property. Values are reported with one digit more than is justified by the experimental uncertainty to avoid round-off errors in calculations based on these results.

[c] Extrapolated values.

Table S11. Standard Thermodynamic Functions of L-Lysine in the Crystal State[a] at p = 0.1 MPa.[b]

T / K	C^{o}_{pm} / J·K^{-1}·mol^{-1}	S^{o}_{m} / J·K^{-1}·mol^{-1}	$\Delta_0^T H^{o}_{m}$ / kJ·mol^{-1}	$\Delta_0^T G^{o}_{m}$ / kJ·mol^{-1}
		crystal III		
1[c]	1.289E−03	3.985E−04	3.035E−07	−9.500E−08
2	1.325E−02	3.933E−03	6.047E−06	−1.818E−06
3	5.298E−02	1.557E−02	3.605E−05	−1.065E−05
4	1.383E−01	4.123E−02	1.273E−04	−3.764E−05
5	2.816E−01	8.634E−02	3.321E−04	−9.960E−05
6	4.912E−01	1.551E−01	7.126E−04	−2.182E−04
7	7.779E−01	2.513E−01	1.340E−03	−4.190E−04
8	1.150	3.785E−01	2.297E−03	−7.312E−04
9	1.610	5.396E−01	3.669E−03	−1.187E−03
10	2.159	7.369E−01	5.547E−03	−1.823E−03
11	2.795	9.718E−01	8.017E−03	−2.674E−03
12	3.511	1.245	1.116E−02	−3.779E−03
13	4.302	1.557	1.506E−02	−5.177E−03
14	5.159	1.907	1.979E−02	−6.906E−03
15	6.076	2.294	2.540E−02	−9.003E−03
16	7.045	2.716	3.196E−02	−1.151E−02
17	8.059	3.174	3.951E−02	−1.445E−02
18	9.113	3.664	4.809E−02	−1.786E−02
19	10.20	4.186	5.774E−02	−2.179E−02
20	11.33	4.738	6.851E−02	−2.625E−02
25	17.34	7.901	1.399E−01	−5.757E−02
30	23.69	11.62	2.425E−01	−1.062E−01
35	30.04	15.75	3.769E−01	−1.745E−01
40	36.19	20.17	5.425E−01	−2.642E−01
45	42.06	24.77	7.383E−01	−3.765E−01
50	47.63	29.49	9.626E−01	−5.121E−01
55	52.96	34.29	1.214	−6.715E−01
60	58.04	39.11	1.492	−8.550E−01
65	62.90	43.95	1.794	−1.063
70	67.54	48.78	2.120	−1.295
75	71.97	53.60	2.469	−1.551
80	76.22	58.38	2.840	−1.830
85	80.30	63.12	3.231	−2.134
90	84.22	67.82	3.643	−2.462
95	88.00	72.48	4.073	−2.812
100	91.66	77.09	4.522	−3.186
110	98.65	86.15	5.474	−4.003
120	105.3	95.02	6.494	−4.909
130	111.7	103.7	7.579	−5.903
140	117.9	112.2	8.727	−6.982
150	124.0	120.6	9.937	−8.146
160	130.0	128.8	11.21	−9.393
170	136.1	136.8	12.54	−10.72
180	142.2	144.8	13.93	−12.13
190	148.5	152.6	15.38	−13.62

T / K	C^{o}_{pm} / J·K^{-1}·mol^{-1}	S^{o}_{m} / J·K^{-1}·mol^{-1}	$\Delta_0^T H^{o}_{m}$ / kJ·mol^{-1}	$\Delta_0^T G^{o}_{m}$ / kJ·mol^{-1}
200	154.9	160.4	16.90	−15.18
210	161.5	168.1	18.48	−16.82
220	168.4	175.8	20.13	−18.54
230	175.7	183.4	21.85	−20.34
240	183.3	191.1	23.65	−22.21
250	192.4	198.7	25.52	−24.16
260	205.0	206.5	27.51	−26.19
270	223.4	214.6	29.64	−28.29
273.15	231.0	217.2	30.36	−28.97
280	251.3	223.2	32.01	−30.48
290	294.6	232.7	34.72	−32.76
291.1 [c]	300.7	233.8	35.05	−33.01
		crystal II		
291.1 [c]	300.5	233.8	35.05	−33.01
298.15	289.2	240.7	37.09	−34.69
300	287.2	242.5	37.62	−35.13
310	281.9	251.8	40.46	−37.60
320	284.2	260.8	43.29	−40.17
330	292.6	269.7	46.17	−42.82
340	305.9	278.6	49.16	−45.56
350	323.6	287.7	52.30	−48.39
360	344.9	297.1	55.64	−51.32
370	369.0	306.9	59.21	−54.34
370.3 [c]	369.8	307.2	59.32	−54.43
		crystal I		
370.3 [c]	290.4	315.7	62.49	−54.43
380	294.8	323.0	65.22	−57.52
390	299.1	330.7	68.19	−60.79
400	303.3	338.3	71.20	−64.14
410	307.3	345.9	74.25	−67.56
420	311.2	353.3	77.34	−71.06

[a] Crystal structure deposited in the Cambridge Structural Database with refcode CUFFUG was reported without specification of temperature and probably corresponds to crystal III or crystal II (see the section 2.2 and Table 2 in the main article).

[b] The combined expanded uncertainty of heat capacity $U_c(C^{o}_{pm})$ as well as of all calculated thermodynamic values (with 0.95 level of confidence, k=2) is: $U_c(X)$=0.1 X below 10 K; $U_c(X)$=0.03 X in temperature range (10 to 40) K; $U_c(X)$=0.02 X in temperature range (40 to 260) K; $U_c(X)$=0.01 X in temperature range (260 to 350) K; $U_c(X)$=0.02 X in temperature range (350 to 420) K, where X represents the heat capacity or the thermodynamic property. Values are reported with one digit more than is justified by the experimental uncertainty to avoid round−off errors in calculations based on these results.

[c] Extrapolated values.

Table S12. Standard Thermodynamic Functions of L-Methionine in the Crystal State[a] at p = 0.1 MPa.[b]

T / K	C^{o}_{pm} / J·K^{-1}·mol^{-1}	S^{o}_{m} / J·K^{-1}·mol^{-1}	$\Delta_0^T H^{o}_{m}$ / kJ·mol^{-1}	$\Delta_0^T G^{o}_{m}$ / kJ·mol^{-1}
		crystal IV (refcode LMETON10)[a]		
1[c]	2.327E−03	7.756E−04	5.818E−07	−1.939E−07
2[c]	1.859E−02	6.203E−03	9.304E−06	−3.102E−06
3[c]	6.245E−02	2.089E−02	4.697E−05	−1.569E−05
4[c]	1.468E−01	4.929E−02	1.477E−04	−4.945E−05
5[c]	2.835E−01	9.567E−02	3.581E−04	−1.203E−04
6[c]	4.828E−01	1.640E−01	7.356E−04	−2.481E−04
7[c]	7.534E−01	2.577E−01	1.348E−03	−4.567E−04
8[c]	1.102	3.802E−01	2.268E−03	−7.731E−04
9[c]	1.533	5.341E−01	3.579E−03	−1.228E−03
10[c]	2.050	7.216E−01	5.364E−03	−1.853E−03
11[c]	2.654	9.446E−01	7.708E−03	−2.683E−03
12	3.343	1.204	1.070E−02	−3.754E−03
13	4.115	1.502	1.442E−02	−5.104E−03
14	4.967	1.838	1.896E−02	−6.771E−03
15	5.893	2.211	2.438E−02	−8.792E−03
16	6.889	2.623	3.077E−02	−1.121E−02
17	7.946	3.072	3.818E−02	−1.405E−02
18	9.059	3.558	4.668E−02	−1.736E−02
19	10.22	4.078	5.631E−02	−2.118E−02
20	11.42	4.633	6.713E−02	−2.553E−02
25	17.82	7.861	1.401E−01	−5.647E−02
30	24.37	11.69	2.456E−01	−1.051E−01
35	30.67	15.93	3.833E−01	−1.741E−01
40	36.64	20.41	5.517E−01	−2.648E−01
45	42.35	25.06	7.493E−01	−3.785E−01
50	47.80	29.81	9.748E−01	−5.156E−01
55	52.99	34.61	1.227	−6.766E−01
60	57.92	39.43	1.504	−8.617E−01
65	62.59	44.26	1.806	−1.071
70	67.03	49.06	2.130	−1.304
75	71.24	53.83	2.476	−1.562
80	75.24	58.55	2.842	−1.842
85	79.06	63.23	3.228	−2.147
90	82.71	67.85	3.632	−2.475
95	86.21	72.42	4.055	−2.825
100	89.57	76.93	4.494	−3.199
110	95.94	85.77	5.422	−4.012
120	101.9	94.37	6.412	−4.913
130	107.6	102.8	7.460	−5.899
140	113.1	110.9	8.564	−6.968
150	118.5	118.9	9.722	−8.117
160	123.8	126.8	10.93	−9.346
170	129.1	134.4	12.20	−10.65
180	134.4	141.9	13.52	−12.03
190	139.9	149.4	14.89	−13.49

T / K	C^{o}_{pm} / J·K^{-1}·mol^{-1}	S^{o}_{m} / J·K^{-1}·mol^{-1}	$\Delta_0^T H^{o}_{m}$ / kJ·mol^{-1}	$\Delta_0^T G^{o}_{m}$ / kJ·mol^{-1}
200	145.4	156.7	16.31	−15.02
210	151.2	163.9	17.80	−16.62
220	157.2	171.1	19.34	−18.30
230	163.7	178.2	20.94	−20.04
240	171.4	185.3	22.62	−21.86
250	180.9	192.5	24.38	−23.75
260	193.0	199.8	26.24	−25.71
270	208.9	207.4	28.25	−27.75
273.15	214.8	209.9	28.92	−28.41
280	229.9	215.4	30.44	−29.86
290	258.2	223.9	32.87	−32.06
298.15	288.9	231.5	35.09	−33.91
300	297.1	233.3	35.64	−34.34
308.4 [c]	341.7	242.0	38.31	−36.34
crystal III (refcode LMETON13)[a]				
308.4 [c]	340.9	242.0	38.31	−36.34
310	321.1	243.8	38.84	−36.73
320	256.0	252.7	41.66	−39.21
325	244.6	256.6	42.91	−40.48
330	240.3	260.3	44.12	−41.78
340	242.9	267.5	46.52	−44.41
350	248.3	274.6	48.98	−47.13
360	253.0	281.7	51.49	−49.91
370	258.4	288.7	54.04	−52.76
380	265.7	295.6	56.66	−55.68
390	276.5	302.7	59.37	−58.67
393.0 [c]	280.6	304.8	60.20	−59.58
crystal II (refcode LMETON14)[a]				
393.0 [c]	272.4	305.1	60.33	−59.58
400	271.2	309.9	62.24	−61.73
410	270.2	316.6	64.94	−64.87
420	270.1	323.1	67.64	−68.07
421.0 [c]	270.1	323.8	67.91	−68.39
crystal I				
421.0 [c]	270.1	323.9	67.95	−68.39
430	270.6	329.6	70.39	−71.33
440	271.9	335.8	73.10	−74.66
450	273.9	341.9	75.83	−78.05
460	276.4	348.0	78.58	−81.50
470	279.6	354.0	81.36	−85.01

[a] Crystal structure deposited in the Cambridge Structural Database; see also Table 2 in the main article.

[b] The combined expanded uncertainty of heat capacity $U_c(C^{o}_{pm})$ as well as of all calculated thermodynamic values (with 0.95 level of confidence, k=2) is: $U_c(X)$=0.1 X below 10 K; $U_c(X)$=0.03 X in temperature range (10 to 40) K; $U_c(X)$=0.02 X in temperature range (40 to 260) K; $U_c(X)$=0.01 X in temperature range (260 to 350) K; $U_c(X)$=0.02 X in temperature range (350 to 430) K, where X represents the heat capacity or the thermodynamic property. Values are reported with one digit more than is justified by the experimental uncertainty to avoid round−off errors in calculations based on these results.

[c] Extrapolated values.

References

1. Varushchenko, R.M.; Druzhinina, A.I.; Sorkin, E.L. Low-temperature heat capacity of 1-bromoperfluorooctane. *J. Chem. Thermodyn.* **1997**, *29*, 623-637, doi:https://doi.org/10.1006/jcht.1996.0173.
2. Lukyanova, V.A.; Druzhinina, A.I.; Pimenova, S.M.; Ioutsi, V.A.; Buyanovskaya, A.G.; Takazova, R.U.; Sagadeyev, E.V.; Gimadeev, A.A. Thermodynamic properties of l-tryptophan. *J. Chem. Thermodyn.* **2017**, *105*, 44-49, doi:https://doi.org/10.1016/j.jct.2016.09.041.
3. Lukyanova, V.A.; Druzhinina, A.I.; Pimenova, S.M.; Ioutsi, V.A.; Buyanovskaya, A.G.; Takazova, R.U.; Sagadeev, E.V.; Gimadeev, A.A. Thermodynamic properties of l-threonine. *J. Chem. Thermodyn.* **2018**, *116*, 248-252, doi:https://doi.org/10.1016/j.jct.2017.09.022.
4. Cole, A.G.; Hutchens, J.O.; Stout, J.W. Heat capacities from 11 to 305°K. and entropies of L-phenylalanine, L-proline, L-tryptophan, and L-tyrosine. Some free energies of formation. *J. Phys. Chem.* **1963**, *67*, 1852-1855, doi:https://doi.org/10.1021/j100803a027.
5. Pokorný, V.; Štejfa, V.; Havlín, J.; Růžička, K.; Fulem, M. Heat Capacities of l-Histidine, l-Phenylalanine, l-Proline, l-Tryptophan and l-Tyrosine. *Molecules* **2021**, *26*, 4298, doi:https://doi.org/10.3390/molecules26144298.
6. Deiko, Y.A.; Il'in, D.Y.; Druzhinina, A.I.; Konstantinova, N.M.; Lukonina, N.S.; Dmitrienko, A.O.; Lysenko, K.A.; Tarazanov, S.V.; Luk'yanova, V.A. Thermodynamic Properties of L-Asparagine Monohydrate. *Russ. J. Phys. Chem. A* **2022**, *96*, 1840-1848, doi:https://doi.org/10.1134/S0036024422090060.
7. Hutchens, J.O.; Cole, A.G.; Robie, R.A.; Stout, J.W. Heat capacities from 11 to 305°K, entropies and free energies of formation of L-asparagine monohydrate, L-aspartic acid, L-glutamic acid, and L-glutamine. *J. Biol. Chem.* **1963**, *238*, 2407-2412, doi:https://doi.org/10.1016/S0021-9258(19)67985-8.
